# Comparison of organ volumes and standardized uptake values in [18F]FDG‐PET/CT images using MOOSE and TotalSegmentator to segment CT images

**DOI:** 10.1002/mp.70025

**Published:** 2025-09-24

**Authors:** Julie Auriac, Christophe Nioche, Narinée Hovhannisyan‐Baghdasarian, Charlotte Loisel, Romain‐David Seban, Nina Jehanno, Lalith Kumar Shiyam Sundar, Thomas Beyer, Irène Buvat, Fanny Orlhac

**Affiliations:** ^1^ LITO U1288 Institut Curie PSL University Inserm Orsay France; ^2^ Institut Curie Department of Nuclear Medicine Paris‐St Cloud France; ^3^ Quantitative Imaging and Medical Physics (QIMP) Team Medical University of Vienna Vienna Austria

**Keywords:** PET/CT, radiomics, segmentation

## Abstract

**Background:**

Manual segmentation of organs from PET/CT images is a time‐consuming and highly operator‐dependent task. Open software solutions are now available to automatically segment all major anatomical structures in CT images.

**Purpose:**

We compared the volumes and standardized uptake values (SUV) extracted from [18F]FDG‐PET/CT patient scans for 33 anatomical structures segmented using two deep learning (DL) algorithms to determine if they are interchangeable.

**Methods:**

Baseline [18F]FDG‐PET/CT images were collected retrospectively for 315 women with metastatic breast cancer. A total of 33 anatomical volumes of interest (VOI) were segmented from the whole‐body CT scans using both MOOSE v.3.0.14 and TotalSegmentator v.2.0.5 and copied onto the corresponding PET images. For each VOI, the volume from the CT image and SUVmax, SUVpeak and SUVmean from the PET image were extracted. The resulting values were compared using the relative difference for each feature.

**Results:**

Following DL segmentation, resulting organ volumes differed by less than 10% for 19/33 organs in more than 80% (252/315) of patients. Four organs were segmented with volume differences greater than 20% in 1/5th of patients: bladder (48%, *p* < 0.0001), portal and splenic veins (34%, *p* < 0.0001), thyroid (16%, *p* < 0.0001), adrenal glands (15%, *p* < 0.0001). SUVmax and SUVpeak were affected by the choice of DL algorithms, with values differing by less than 10% in more than 80% of patients for only 16 and 19 out of 33 organs, respectively. In contrast, SUVmean was less affected with differences of less than 10% in more than 80% of patients for all anatomical structures, except the bladder, lungs and skull.

**Conclusions:**

The two software tools produce similar results in volume estimates for most anatomical structures. SUVmean is less dependent on the segmentation algorithm than SUVmax and SUVpeak and shows excellent reproducibility for all anatomical structures studied except for the bladder, the lungs and the skull.

## 1 INTRODUCTION

The segmentation of lesions, organs or tissues is key in medical image analysis. The delineation of regions of interest (ROIs) facilitates the extraction of biomarkers, whose values and progression during treatment can provide critical insights for patient management.^1^, ^2^, ^3^ While manual expert‐delineation is often considered the “reference”, it is time‐consuming, labor‐intensive and highly operator‐dependent. With the development of deep learning for image analysis, algorithms trained on large datasets can now successfully segment medical images fast and with great accuracy, both for lesions, such as tumor foci and healthy organs/tissues.^4^, ^5^

In nuclear medicine, 18F‐fluorodeoxyglucose ([18F]FDG) positron emission tomography/computed tomography (PET/CT) imaging is widely used for oncology staging and treatment monitoring. Many research studies are conducted to determine whether image‐based features derived from tumor regions could help to refine tumor characterization, assist in prognostication and predict response to treatment.^6^, ^7^ Some recent studies also suggest that the metabolic activity in various organs and tissue measured from [18F]FDG images across the entire body can provide valuable information for predicting cancer patient outcome^8^, ^9^, ^10^, ^11^, ^12^ and understanding the underlying pathophysiological mechanisms is an area of active investigation in translational research.

Today, several organ‐based segmentation algorithms applicable to CT images are available.^13^, ^14^, ^15^, ^16^ Multiple‐Organ Objective Segmentation (MOOSE)^17^ and TotalSegmentator^18^ are two open‐access deep learning algorithms that provide an automated segmentation of a large number of anatomical structures from CT images. Initiatives are emerging within the scientific community to assess the quality and discrepancies in segmentations produced by these software tools, including as part of the NA‐MIC Project Weeks (https://projectweek.na‐mic.org/PW42_2025_GranCanaria/Projects/ReviewOfSegmentationResultsQualityAcrossVariousMultiOrganSegmentationModels/). In this context, we investigated how the volumes and standardized uptake values (SUV) extracted from 33 anatomical structures of whole‐body [18F]FDG‐PET/CT images differed depending on whether MOOSE and TotalSegmentator was used for segmentation to determine whether they could be used interchangeably.

## 2 MATERIALS AND METHODS

### 2.1 Data

Our dataset included baseline whole‐body [18F]FDG‐PET/CT images from 315 women with confirmed metastatic breast cancer. Image acquisition was performed between 2009 and 2019 before any treatment and using different PET/CT systems (Table S1). For PET, the [18F]FDG injected dose was 242 ± 71 MBq, the injection‐to‐acquisition time was 63 ± 10 min, with data acquisition of 2.0 ± 0.6 min per bed position. PET images were expressed in SUV units normalized by the patient body weight.

This study was approved by the institutional review board of Institut Curie (DATA220207). Informed consent was obtained for all patients through institutional processes. All related data were de‐identified, collected and stored in compliance with GDPR.

### 2.2 Image analysis

#### 2.2.1 Software

Volumes of interest (VOIs) corresponding to anatomical regions were automatically segmented on the whole‐body CT images with MOOSE v.3.0.14 and TotalSegmentator v.2.0.5 software, both based on a model from the nnU‐net framework. The main characteristics of each software are summarised in Table 1.

**TABLE 1:** Key features and requirements for MOOSE and TotalSegmentator.

Software	Date of download	Version	Method	Training datasets	Number of classes	System requirements
MOOSE (https://github.com/ENHANCE‐PET/MOOSE)	April 17, 2025	v3.0.14	nnU‐Net	1683 CT (655 F/1028 M)	120	16 GB of RAM
TotalSegmentator (https://github.com/wasserth/TotalSegmentator)	May 23, 2024	v2.0.5	nnU‐Net	1559 CT (≈500 F/700 M	>117	7.4 GB of RAM

In MOOSE software,^17^ a total of 1,683 CT scans acquired from 655 females and 1,028 males using multiple CT systems and imaging protocols at different imaging sites were used for training. Among them, 542 were acquired from healthy controls, 43 from arthritis patients and 1,098 from oncology patients. MOOSE v.3.0.14 provides the segmentation of all major anatomic structures from the whole‐body with a total of 120 classes in open access.

In TotalSegmentator (TS) software,^18^ a total of 1,559 CT scans acquired from around 500 females and 700 males with multiple CT systems and imaging protocols at different imaging sites were used for the training. TotalSegmentator v.2.0.5 offers the segmentation of all major anatomic structures across the entire body, with a total of 117 classes available in open access. For faster runtime and less memory requirement, the “fast” option can be used by running the model with the lower resolution. Other segmentations, such as that of skeletal muscles and adipose tissues, are also available by requesting a free licence. Of note, the “fast” option is not available for these additional segmentations.

The correspondence between model names and VOIs in MOOSE and TotalSegmentator is given in Table S2 of the Supplementary Material. TotalSegmentator was run with the fast option when available, unless otherwise stated in the results section. The study was conducted using Linux Ubuntu 20.04.3 with 256 Gb of RAM memory, an Intel Xeon Gold 5218R 64 bit, and a 16 Gb Nvidia Quadro RTX 5000 graphic card.

#### 2.2.2 Features extraction

**FIGURE 1:**
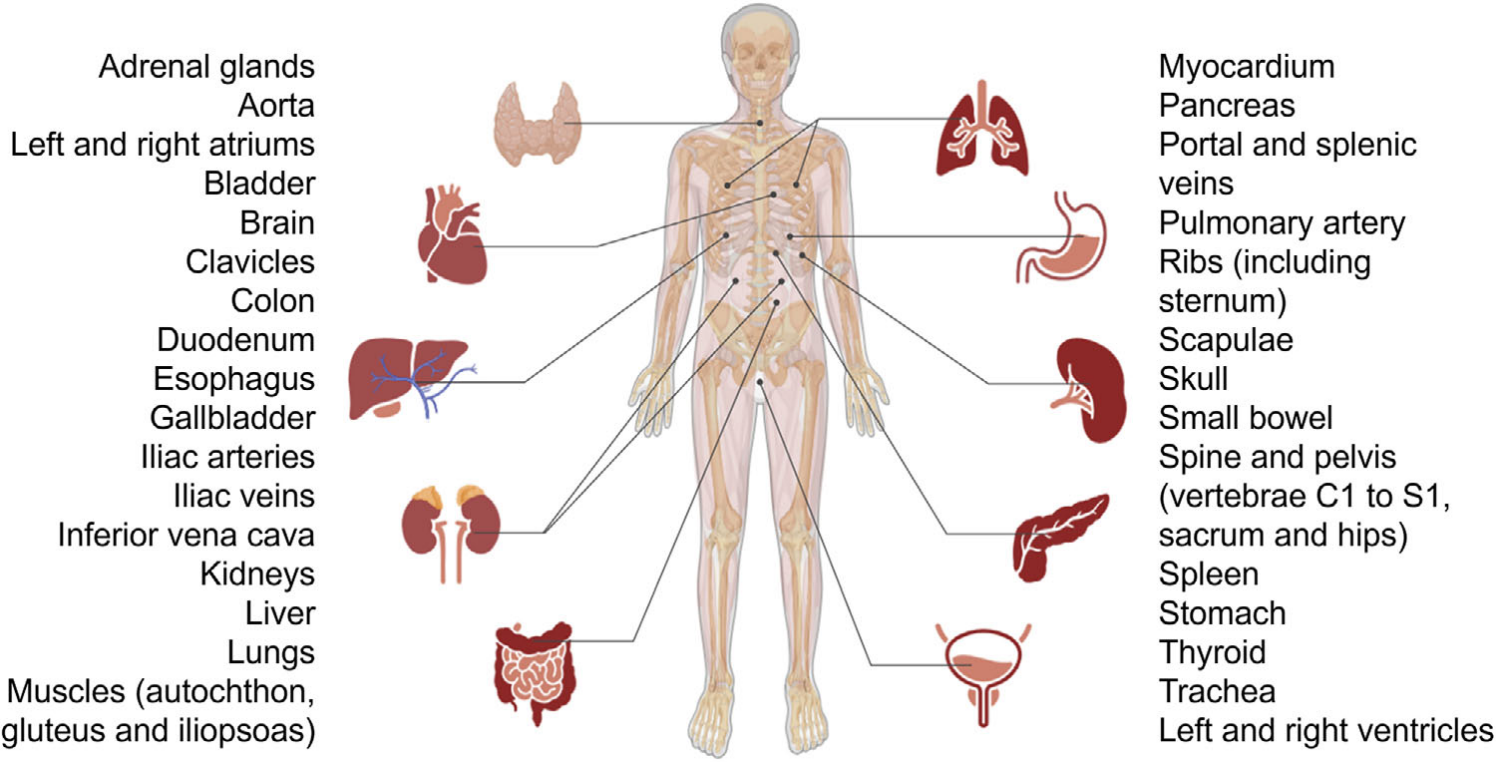
List of all 33 common anatomic structures segmented by TotalSegmentator and MOOSE used for our study.

After the segmentation of all the anatomic structures by TotalSegmentator and MOOSE, we selected 33 regions available in both software, listed in Figure 1 and in Table S2. Since the patients have metastases, some regions may contain tumor lesions yet this does not invalidate the comparison between the two software. We also studied subcutaneous fat segmented with TotalSegmentator (available only without the fast option) for the whole‐body and in MOOSE only for the third lumbar vertebrae region. All PET and CT images, as well as segmentation masks, were resampled to a fixed 2 × 2 × 2 mm^3^ voxel size to calculate the volumes and SUVs using LIFEx freeware (v.7.7.3,^19^ www.lifexsoft.org). The total volume in millilitres (mL) of the anatomic structure was measured on the CT images. Within that structure, the maximum SUV (SUVmax), the mean SUV measured within the 1 cm3 spherical volume in which it was the highest (SUVpeak) and average SUV (SUVmean) in the VOI were computed from the PET images associated with the CT.

### 2.3 Statistical analysis

To evaluate the similarity of the segmentation masks resulting from the two algorithms, we calculated the Dice Similarity Coefficient (DSC) and Hausdorff distance for each anatomical structure and each patient. DCS greater than 0.7 was considered excellent.

Regarding Volume and SUV features, the agreement between the software results for each patient (p) and each VOI (i) was evaluated by calculating the difference:

$$\begin{array}{cc} \Delta Feature(p,i) & =Feature(p,i)MOOSE\;\\ & \;-\;Feature\;(p,i)TS \end{array}$$

and the relative difference:

$$\begin{array}{cc} \Delta relFeature(p,i) & =2\;*\;|Feature(p,i)MOOSE\;\\ & \;-\;Feature\;(p,i)TS|\;/\;(Feature(p,i)\\ & MOOSE+Feature(p,i)TS)\;*\;100 \end{array}$$

We classified each anatomical structure and each feature into three groups according to their reproducibility based on ∆_rel_Feature (Table 2). In cases where no segmentation was generated by either algorithm, the patient was excluded from the analysis for the corresponding anatomic structure. Reproducibility of SUVpeak was evaluated only when SUVpeak values were successfully computed in more than 50% of patients by both algorithms, due to the volume of 1cm3 required for SUVpeak calculation.

**TABLE 2:** Classification of anatomical structures according to reproducibility between software tools based on volume, SUVmax, SUVpeak and SUVmean.

Level of reproducibility	Feature	Proportion of patients (%)
High	∆_rel_ ≤ 10%	≥80%
Moderate	∆_rel_ ≤ 20%	≥80%
Poor	∆_rel_ > 20%	≥20%

For subcutaneous fat, as TotalSegmentator estimates it from the whole‐body scan and MOOSE from the L3 vertebra segmentation, only SUV values were analyzed to assess the reproducibility between the two algorithms.

A paired sample Wilcoxon test was used to determine if the feature values Volume, SUVmax, SUVpeak and SUVmean differed significantly between the two segmentations, with p‐values lower than 0.05 interpreted as statistically significant. We also calculated the Spearman's rank correlation coefficient (rS) between the values calculated from the two algorithms.

All statistical analyses were performed using R version 4.2.2 and python 3.11.7.

## 3 RESULTS

The mean age and body mass index (BMI) of the study population were 54 ± 14 years (range: 23–89 years) and 25.8 ± 5.4 kg/m^2^ (range: 15.7 – 53.3 kg/m^2^) respectively. The patients’ mean weight was 68 ± 15 kg (range: 41 – 140 kg), and their mean height was 1.63 ± 0.07 m (range: 1.42 – 1.84 m). Running MOOSE was faster than TotalSegmentator for the segmentation of the 33 common anatomical structures and subcutaneous fat (373s and 601s in average per scan with GPU, respectively with our configuration). When using TotalSegmentator's fast option, the segmentation runtime was 235s per scan.

The concordance between the two algorithms was excellent for all anatomic structures according to the DSC (Table S3) with values higher than 0.7 except for adrenal glands (average DSC: 0.69 ± 0.11), portal and splenic veins (0.55 ± 0.22) and the right ventricle (0.69 ± 0.11). The average Hausdorff distance ranged from 5.28 ± 1.64 mm for the left atrium and 176.85 ± 110.87 mm for pulmonary artery.

Table 3 summarizes the differences observed between feature values extracted from the MOOSE and TotalSegmentator (fast option) segmentations. A detailed comparison between MOOSE and TotalSegmentator feature values extracted from each of the 33 VOI is provided in Supplemental Table S4. No major difference was observed between feature values calculated with and without TotalSegmentator's fast option (Tables S4 and S5).

**TABLE 3:** Summary of the differences observed between MOOSE and TotalSegmentator (with fast option when available) for the 33 volumes of interest (VOI).

Feature	Number of VOI with *p* < 0.05	rS [min–max]	Mean ± 1 standard deviation of the absolute difference by VOI
Volume	28/33	[0.66^a^–1.00]	Min = 0 ± 1 mL for trachea Max = 47 ± 11 mL for ribs
SUVmax	23/33	[0.78^b^–1.00]	Min = 0.0 ± 0.1 SUV for brain Max = 3.1 ± 9.1 SUV for kidneys
SUVpeak^*^	15/28	[0.84^c^–1.00]	Min = 0.0 ± 0.2 SUV for brain Max = 0.7 ± 1.0 SUV for skull
SUVmean	16/33	[0.77^a^–1.00]	Min = 0.00 ± 0.04 SUV for left atrium Max = 4.75 ± 5.98 SUV for bladder

p: paired samples Wilcoxon test; rS: Spearman correlation coefficients; ∆X(p,i) = |X(p,i)_MOOSE_—X(p,i)_TS_| with TS = TotalSegmentator, X = feature, p = patient and i = VOI.

^a^ Bladder.

^b^ Small bowel.

^c^ Skull.

* Reproducibility was evaluated only for 28 structures, i.e., when SUVpeak was successfully computed in more than 50% of patients by both algorithms.

The Volume estimates were highly reproducible between the two software for 19 anatomical structures (Table 4), with a difference of less than 10% in at least 80% of cases. However, the Volume estimates were poorly reproduced for four anatomical regions (adrenal glands, bladder, portal and splenic veins, and thyroid), with a difference of at least 20% in at least 20% of patients. The differences between MOOSE and TotalSegmentator ranged from ‐4 to 6 mL in the adrenal glands, ‐346 to 56 mL in the bladder, −6 to 4 mL in the portal and splenic veins, and −23 to 8 mL in the thyroid (Table S4). The Volumes were significantly different for 28/33 anatomical structures between the two software (Tables 3 and Table S4): on average, 16 VOIs had a smaller volume when segmented by MOOSE compared to TotalSegmentator. The largest differences were observed for bladder (48%, *p* < 0.0001), portal and splenic veins (34%, *p*< 0.0001), thyroid (16%, *p* < 0.0001) and adrenal glands (15%, *p* < 0.0001). The correlation between Volumes values was excellent (rS ≥ 0.87), except for bladder (rS = 0.66).

**TABLE 4:** Evaluation of the impact of the segmentation method (MOOSE and TotalSegmentator) on the Volume, SUVmax, SUVpeak and SUVmean of 33 anatomical structures of interest.

VOI	Volume (mL)	SUVmax	SUVpeak	SUVmean
Adrenal glands	⨯	✔	NA	✔
Aorta	✔	▲	✔	✔
Atrium L	▲	▲	✔	✔
Atrium R	▲	✔	✔	✔
Bladder	⨯	✔	✔	⨯
Brain	✔	✔	✔	✔
Clavicles	✔	✔	✔	✔
Colon	✔	▲	✔	✔
Duodenum	▲	▲	✔	✔
Esophagus	✔	✔	▲	✔
Gallbladder	▲	✔	▲	✔
Iliac arteries	▲	▲	NA	✔
Iliac veins	✔	▲	▲	✔
Inferior vena cava	▲	✔	✔	✔
Kidneys	✔	▲	▲	✔
Liver	✔	▲	✔	✔
Lungs	✔	▲	▲	▲
Muscles	✔	⨯	▲	✔
Myocardium	✔	✔	NA	✔
Pancreas	▲	✔	✔	✔
Portal & Splenic veins	⨯	✔	NA	✔
Pulmonary artery	▲	▲	✔	✔
Ribs	▲	▲	✔	✔
Scapulae	✔	✔	✔	✔
Skull	✔	▲	⨯	▲
Small bowel	✔	⨯	▲	✔
Spine and Pelvis	✔	⨯	✔	✔
Spleen	✔	▲	✔	✔
Stomach	✔	✔	✔	✔
Thyroid	⨯	✔	NA	✔
Trachea	✔	▲	▲	✔
Ventricle L	✔	✔	✔	✔
Ventricle R	▲	✔	✔	✔

✔: high reproducibility (∆_rel_(Feature) ≤ 10% for at least 80% of patients).

▲: moderate reproducibility (∆_rel_(Feature) ≤ 20% for at least 80% of patients).

⨯: poor reproducibility (∆_rel_(Feature) > 20% for at least 20% of patients).

NA: reproducibility was evaluated only for 28 structures, i.e., when SUVpeak was successfully computed in more than 50% of patients by both algorithms.

**FIGURE 2:**
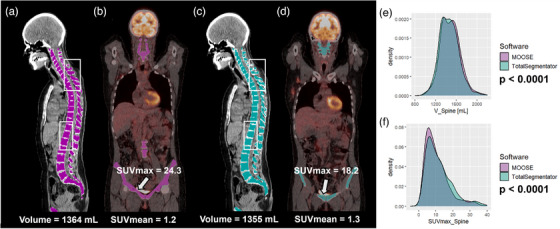
Analysis of the spine and pelvis segmented by MOOSE and TotalSegmentator. (a–d) [18F]FDG PET/CT scans of one patient with spine and pelvis segmented by MOOSE (in purple) and TotalSegmentator (in blue). The white box highlights the mismatch between the segmentations. e–f. Distribution of spine and pelvis volume (in mL) and SUVmax for the two algorithms for all patients.

**FIGURE 3:**
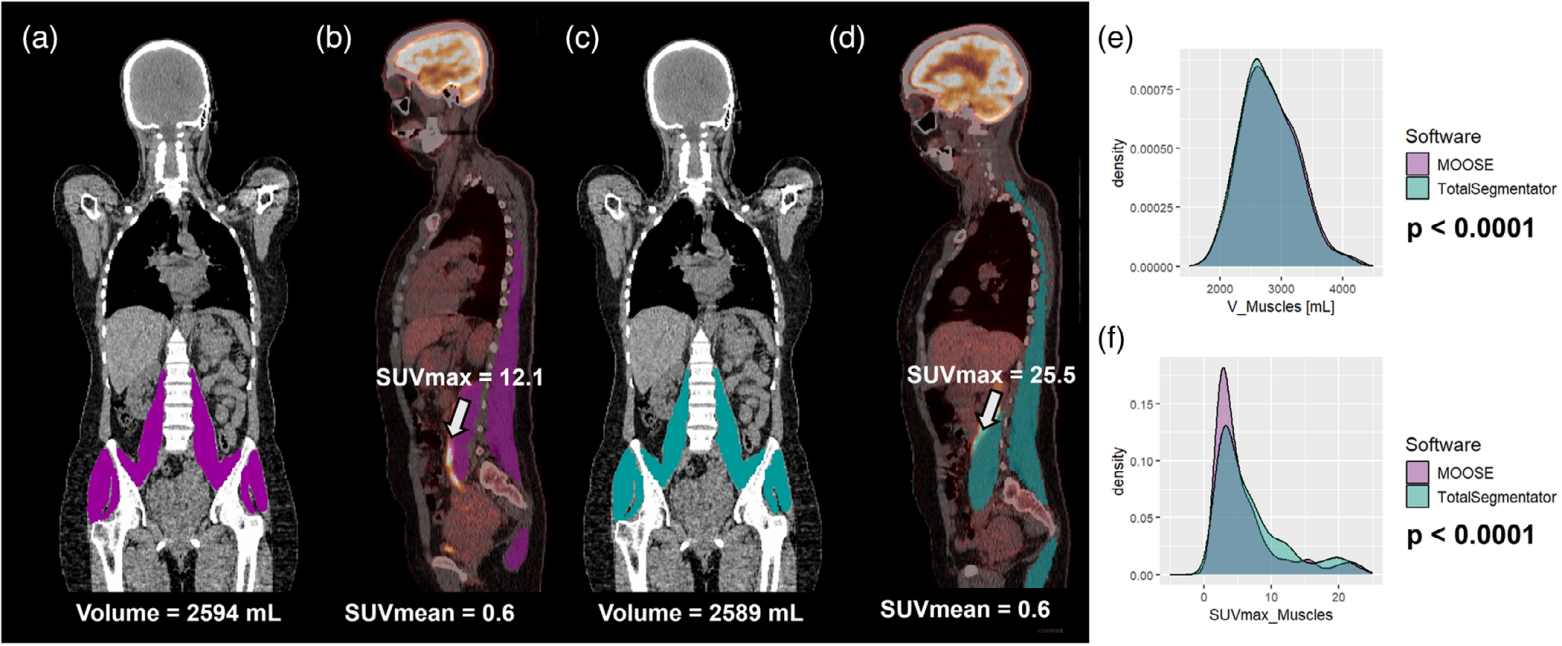
Analysis of a group of 10 muscles segmented by MOOSE and TotalSegmentator. (a–d) 18F FDG PET/CT scans of one patient with muscles segmented by MOOSE (in purple) and TotalSegmentator (in blue). e–f. Distribution of muscle volume (in mL) and SUVmax for the two algorithms for all patients.

The SUVmax values were moderately reproducible between the two algorithms for 14 annotated structures with a difference of less than 20% in more than 80% of patients (Table 4). SUVmax values were poorly reproducible for three regions (muscles, small bowel and spine) with a difference of more than 20% for at least 20% of patients. A significant difference in SUVmax was observed for 23 VOIs (Tables 3, S4 and S5). The largest differences were observed for muscles (average difference of 28%, *p* < 0.0001), small bowel (23%, *p* = 0.04) and spine and pelvis (17%, *p* < 0.0001). As an example, while the estimation of the muscles and spine and pelvis volumes were highly reproducible between the two software (average difference of 1% and 2% respectively), the SUVmax varied by more than 20% in more than 20% of patients depending on the segmentation algorithm for these two compartments (Figures 2 and 3). All anatomical structures exhibited a correlation coefficient higher than 0.83, except for the small bowel (rS = 0.78).

The SUVpeak values were highly reproducible between the two algorithms for 19/28 anatomical structures, with a difference of less than 10% in at least 80% of cases. SUVpeak was significantly different for 15/28 anatomical structures (Tables 3, S4 and S5), but highly correlated between the two algorithms for all VOIs (rS ≥ 0.84).

The SUVmean values were highly reproducible between the two algorithms for 30 anatomical structures. The bladder was the most affected by the choice of algorithms with a SUVmean difference of more than 20% in 52% of patients and a mean difference of (4.8 ± 6.0) SUV (range: [−10.6; 45.9]) for all patients. The lungs and skull exhibited a moderated reproducibility between the two algorithms with a SUVmean difference of more than 20% in 4% of patients for both regions. SUVmean was significantly different for 16 anatomical structures (Tables 3, S4, and S5), but highly correlated between the two algorithms for all VOIs (rS ≥ 0.77).

**FIGURE 4:**
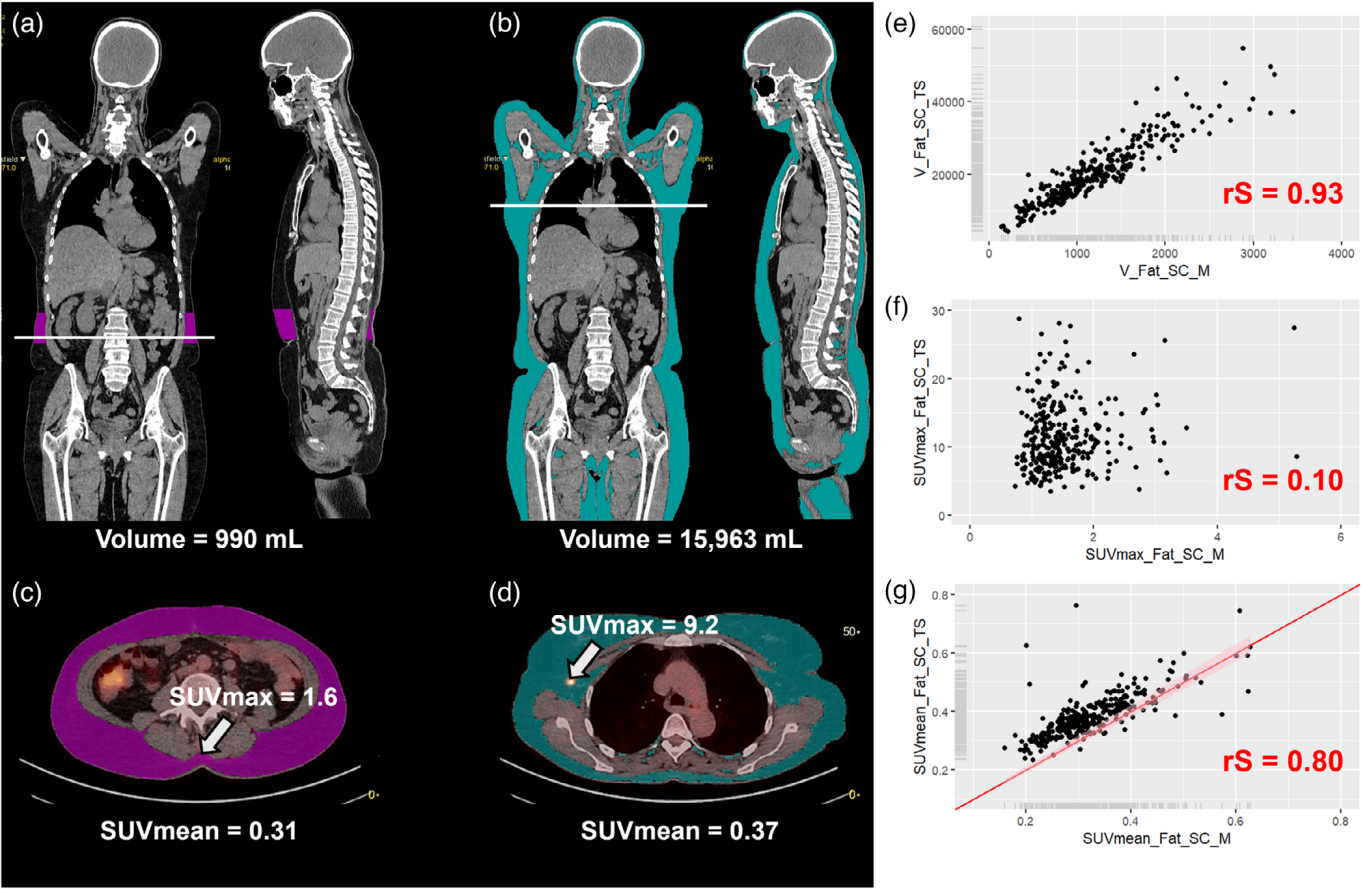
Analysis of the subcutaneous fat segmented by MOOSE (L3 area) and TotalSegmentator (whole‐body). (a–d) 18F FDG PET/CT scans of one patient with subcutaneous fat segmented by MOOSE (in purple, a and c) and TotalSegmentator (in blue, b and d). (e–g) Correlation plot of the subcutaneous fat volume (g), SUVmax (f) and SUVmean (g) between MOOSE and TotalSegmentator. The red line corresponds to the identity line; the pink strip the 5% deviation. rS = Sperman correlation coefficient.

Regarding subcutaneous fat (Figure 4), despite differences in segmentation methods, the correlation between volumes was excellent between the two algorithms (rS = 0.93). SUVmax values obtained between the two software were poorly correlated (rS = 0.10) with differences always exceeding 20%. SUVpeak values were also poorly correlated (rS = 0.30) between the two algorithms with differences exceeding 20% in 99% of cases. SUVmean values were highly correlated (rS = 0.80), with differences exceeding 20% in 52% of cases but only a mean difference of (0.06 ± 0.05) SUV (range: [0.0; 0.5]) for all patients.

## 4 DISCUSSION

Our study aimed to assess whether two publicly available software tools^17^, ^18^ for automated segmentation of anatomical structures based on whole‐body CT images produced comparable results, with a focus on typical [18F]FDG‐PET and CT features calculated from these segmentations. Indeed, a more comprehensive approach to image analysis encompassing both the tumor and healthy organs is essential as it is now well established that a dynamic crosstalk exists between them. The metabolism of these organs as well as their interactions can be altered in the presence of a tumor, potentially influencing disease progression. Moreover, certain organs such as the spleen, liver, and bone marrow, play a critical role in anti‐ and pro‐tumoral immunity. Their metabolic activity and functional state could provide valuable prognostic insights, emphasizing the need to integrate their analysis into oncological assessments.

The comparison of the segmentations produced by the two algorithms using the DSC indicated an overall strong agreement, with a high degree of overlap between the segmentation masks generated by the two algorithms, except for adrenal glands, portal and splenic veins and the right ventricle (Table S3). However, the Hausdorff distance revealed discrepancies in boundary delineation, especially for the pulmonary artery. The discrepancies observed between the two software tools may be due to differences in their training datasets, particularly the heterogeneity in capturing diverse patient characteristics. In particular, MOOSE mostly uses data from healthy individuals or oncology patients, whereas TotalSegmentator relies on CT scans acquired for a broader range of clinical indications.

In our female cohort, the volumes estimated by both segmentation algorithms were in excellent agreement for most subjects and most organs (19/33), with a difference in feature value of less than 20% as observed at least 80% of subjects (Table 4). Our findings reveal that only four anatomical regions—adrenal glands, bladder, portal and splenic veins, and thyroid—showed poor reproducibility of volume estimates between the two algorithms, with a difference of at least 20% in at least 20% of patients. SUVmean measurement was highly reproducible between the two algorithms for all anatomical structures with a difference of less than 10% in at least 80% of patients, except for the bladder, the lungs and the skull. The differences observed between the two algorithms for the lungs are mainly due to the presence of pleural effusion, where MOOSE avoids segmenting the affected lung areas, unlike TotalSegmentator, as shown in Figure S1 of the Supplemental Data. Additionally, in the presence of metastases, MOOSE also appears to exclude lesions on CT corresponding to hotspots in the PET images, which may explain the variations observed in SUV values. Moreover, despite differences in subcutaneous fat segmentation between the two software, the SUVmean value remained highly reproducible between both algorithms, unlike the SUVmax and SUVpeak value which were poorly reproducible. In comparison, SUVmax was poorly or moderately reproducible between MOOSE and TotalSegmentator for 17 anatomical structures. Although both algorithms provided highly comparable volume estimates for the muscles and spine and pelvis, the reproducibility of SUVmax values was poor (Figures 2 and 3). This is because SUVmax is measured in a single voxel and is therefore highly sensitive to the possible inclusion of a few voxels from high uptake neighboring structures, such as the brain, heart, kidneys, ureters and bladder. SUVpeak values were highly reproducible between the two algorithms for 19/28 anatomical structures with a difference of less than 20% in at least 80% of patients. Overall, SUVmax was the most affected by the choice of algorithms with only 16/33 structures showing a high level of reproducibility and with three structures (muscles, small bowel and spine and pelvis) showing a poor reproducibility.

With the rise of metabolic activity analysis across the entire body,^8^, ^9^, ^10^, ^11^, ^12^ the segmentation of organs and tissues will increasingly play a prominent role in medical image analysis as the concept of organomics develops.^12^ Therefore, comparing results reported in different studies requires an understanding of a potential bias introduced by the segmentation algorithm. Our results demonstrate that organ‐based SUVmax and SUVpeak remain highly dependent on the segmentation method used and should preferably not be derived from automated unsupervised segmentation, unlike SUVmean which appears to be very reproducible between segmentation algorithms. According to our findings, SUVmean should be preferred to SUVmax or to SUVpeak to characterize the metabolic activity of non‐tumor tissues. However, other not‐so‐frequently‐used features could be more robust than SUVmean, such as SUVmedian defined as the median of SUV in the VOI. We found that SUVmedian yielded excellent reproducibility for the lungs and skull for instance (Table S6) although the use of SUVmedian did not solve the issue of non‐reproducibility in the bladder. Since SUVmax and SUVpeak can still provide useful and distinct information from SUVmean, their measurements based on automatically segmented anatomical regions should be approached with caution. First, it is essential that all patients in the study be segmented using the same clearly specified version of an algorithm to ensure the reproducibility of the results. Second, as these two biomarkers are calculated from a small number of voxels (1 voxel for SUVmax, 1 cm^3^ for SUVpeak), it is strongly recommended to check for extreme values, which may result from the inclusion of physiological signals from adjacent compartments (e.g., the bladder).

A limitation of our study is that our conclusions pertain to female anatomy and cannot be readily extrapolated to male anatomy. We assumed perfect registration between the PET and CT scans, which may not always be the case. Similarly, we did not account for the differences in spatial resolution between CT and PET when copying the CT mask on the PET scans to make measurements. Yet, any bias possibly due to PET/CT misregistration or differences in PET and CT spatial resolution does not invalidate our comparison results as both algorithms were evaluated on the same images in the same conditions. A further limitation relates to the version of the algorithms used. In TotalSegmentator, using more recent versions (>v2.2.0) provides access to new model‐derived structures from CT images, including head and neck muscles, head muscles, head gland cavities, head and neck bones and vessels, oculomotor muscles, hepatic vessels, kidney cysts, lung nodules, and breasts. Yet, these enhancements did not affect the compartments that we have studied. In MOOSE, the latest released versions fixed some issues but did not provide new structures or major improvements compared to MOOSE v3.0.14. Finally, all patients had metastatic breast cancer, which means that the segmented regions may contain tumor lesions and that the metabolic activities measured in these regions are not necessarily representative of the healthy metabolic activities. Last, we did not aim to assess the reliability of the segmentation, which was already assessed in the respective original contributions^17^, ^18^; instead, we sought to assess the impact of a given segmentation algorithm on selected imaging biomarkers that are important for organ and tissue analysis, such as Volume, SUVmean, SUVpeak and SUVmax.

## 5 CONCLUSION

Our study revealed that both segmentation algorithms exhibited strong agreement in volume estimates for most organs and tissues. SUVmean was much less affected by the choice of the automatic segmentation software than SUVmax and SUVpeak and had close values between MOOSE and TotalSegmentator for all anatomical structures studied, except for the bladder, the lungs and the skull.

## CONFLICT OF INTEREST STATEMENT

The authors have no conflicts to disclose. LS and TB are co‐founders of Zenta GmbH.

## DATA SHARING STATEMENT

Data analyzed during the study were provided by a third party. Requests for data should be directed to the provider indicated in the Acknowledgements.

## Supporting information



Supporting information

Supporting information

Supporting information

Supporting information

Supporting information

Supporting information

Supporting information
