# Supplementary material for: Comparison of organ volumes and standardized uptake values in [18F]FDG‐PET/CT images using MOOSE and TotalSegmentator to segment CT images
Source: Med Phys. 2025 Sep 24;52(10):e70025. doi: 10.1002/mp.70025 (PMC12460933; doi:10.1002/mp.70025)
Supplement: Supplementary file 2 — Supporting information [file MP-52-0-s004.docx]

Table S-2. Anatomical structure correspondence between MOOSE and TotalSegmentator

| **Anatomic structure** | **Model in Moose** | **Name in Moose** | **Model in TotalSegmentator** | **Name in TotalSegmentator** |
| --- | --- | --- | --- | --- |
| Adrenal glands | Clin_ct_organs | 1:adrenal_gland_left, 2:adrenal_gland_right | Total | 8:adrenal_gland_right, 9:adrenal_gland_right |
| Aorta | Clin_ct_organs | 6:aorta | Total | 52:aorta |
| Atrium L | Clin_ct_cardiac | 2:heart_atrium_left | Heartchambers_  highres | 2:atrium_left |
| Atrium R | Clin_ct_cardiac | 3:heart_atrium_right | Heartchambers_  highres | 3:atrium_right |
| Bladder | Clin_ct_organs | 3:bladder | Total | 21:urinary_bladder |
| Brain | Clin_ct_organs | 4:brain | Total | 90:brain |
| Clavicles | Clin_ct_peripheral_  bones | 3:clavicle_left, 4:clavicle_right | Total | 73:clavicula_left, 74:claivula_right |
| Colon | Clin_ct_digestive | 1:colon | Total | 20:colon |
| Duodenum | Clin_ct_digestive | 2:duodenum | Total | 19:duodenum |
| Esophagus | Clin_ct_digestive | 3:esophagus | Total | 15:esophagus |
| Gallbladder | Clin_ct_digestive | 5:gallbladder | Total | 4:gallbladder |
| Iliac arteries | Clin_ct_cardiac | 7:iliac_artery_left, 8:iliac_artery_right | Total | 65:iliac_artery_left, 66:iliac_artery_right |
| Iliac veins | Clin_ct_cardiac | 9:iliac_vena_left, 10:iliac_vena_right | Total | 67:iliac_vena_left, 68:iliac_vena_right |
| Inferior vena cava | Clin_ct_cardiac | 11:inferior_vena_cava | Total | 63:inferior_vena_cava |
| Kidneys | Clin_ct_organs | 6:kidney_left, 7:kidney_right | Total | 2:kidney_right, 3:kidney_left |
| Liver | Clin_ct_organs | 8:liver | Total | 5:liver |
| Lungs | Clin_ct_organs | 1:lung_upper_lobe_left to 5:lung_lower_lobe_right | Total | 10:lung_upper_lobe_left to 14:lung_lower_lobe_right |
| Muscles | Clin_ct_muscles | 1:autochthon_left to 10:iliopsoas_right | Total | 80:gluteus_maximus_ left to 89:iliopsoas_right |
| Myocardium | Clin_ct_cardiac | 1:heart_myocardium | Heartchambers_  highres | 1:myocardium |
| Pancreas | Clin_ct_organs | 14:pancreas | Total | 7:pancreas |
| Portal and Splenic veins | Clin_ct_cardiac | 12:portal_splenic_vein | Total | 64:portal_vein_  and_splenic_vein |
| Pulmonary artery | Clin_ct_cardiac | 13:pulmonary_artery | Heartchambers_  highres | 7:pulmonary_artery |
| Ribs | Clin_ct_ribs | 1:rib_left_1 to 27:sternum | Total | 92:rib_left_1 to 116:sternum |
| Scapulae | Clin_ct_peripheral_  bones | 21:scapula_left, 22:scapula_right | Total | 71:scapulae_left to 72:scapulae_right |
| Skull | Clin_ct_peripheral_  bones | 23:skull | Total | 91:skull |
| Small bowel | Clin_ct_digestive | 4:small_bowel | Total | 18:small bowel |
| Spine and Pelvis | Clin_ct_vertebrae | 1:vertebra_C1 to 28:sacrum | Total | 25:sacrum to 50:vertebrae_C1 |
| Spleen | Clin_ct_organs | 15:spleen | Total | 1:spleen |
| Stomach | Clin_ct_organs | 16:stomach | Total | 6:stomach |
| Thyroid | Clin_ct_organs | 17:thyroid_left, 18:thyroid_right | Total | 17:thyroid_gland |
| Trachea | Clin_ct_organs | 19:trachea | Total | 16:trachea |
| Ventricle L | Clin_ct_cardiac | 4:heart_ventricle_left | Heartchambers_  highres | 5:ventricle_left |
| Ventricle R | Clin_ct_cardiac | 5:heart_ventricle_right | Heartchambers_  highres | 3:ventricle_right |
| Subcutaneous Fat* | Clin_ct_body_  composition | 2:subcutaneous_fat | Tissue_types | 2:subcutaneous_fat |

*Segmentation of subcutaneous fat differs between MOOSE (L3) and TotalSegmentator (whole-body).
