# Supplementary material for: Comparison of organ volumes and standardized uptake values in [18F]FDG‐PET/CT images using MOOSE and TotalSegmentator to segment CT images
Source: Med Phys. 2025 Sep 24;52(10):e70025. doi: 10.1002/mp.70025 (PMC12460933; doi:10.1002/mp.70025)
Supplement: Supplementary file 3 — Supporting information [file MP-52-0-s001.docx]

Table S-3. Average and standard deviation of Dice Similarity Coefficient (DSC) and Hausdorff Distance between MOOSE and TotalSegmentator with fast option for the common volumes of interest (VOI).

| VOI | DSC | Hausdorff distance (mm) |
| --- | --- | --- |
| Adrenal glands | 0.69 ± 0.11 | 8.02 ± 7.41 |
| Aorta | 0.90 ± 0.03 | 9.21 ± 6.26 |
| Atrium L* | 0.92 ± 0.02 | 5.28 ± 1.64 |
| Atrium R* | 0.90 ± 0.03 | 7.36 ± 2.52 |
| Bladder | 0.71 ± 0.19 | 22.54 ± 17.60 |
| Brain | 0.98 ± 0.01 | 8.00 ± 38.49 |
| Clavicles | 0.85 ± 0.02 | 5.37 ± 2.32 |
| Colon | 0.89 ± 0.04 | 43.30 ± 54.66 |
| Duodenum | 0.79 ± 0.12 | 13.56 ± 9.90 |
| Esophagus | 0.80 ± 0.05 | 9.71 ± 34.32 |
| Gallbladder | 0.81 ± 0.19 | 7.60 ± 7.11 |
| Iliac arteries | 0.73 ± 0.06 | 14.56 ± 8.36 |
| Iliac veins | 0.81 ± 0.04 | 19.09 ± 58.96 |
| Inferior vena cava | 0.82 ± 0.05 | 14.69 ± 9.18 |
| Kidneys | 0.92 ± 0.04 | 9.02 ± 9.89 |
| Liver | 0.96 ± 0.02 | 16.85 ± 26.09 |
| Lungs | 0.96 ± 0.04 | 16.01 ± 39.49 |
| Muscles | 0.93 ± 0.01 | 9.85 ± 7.42 |
| Myocardium | 0.86 ± 0.04 | 7.01 ± 11.07 |
| Pancreas | 0.85 ± 0.07 | 9.78 ± 5.90 |
| Portal and Splenic veins | 0.55 ± 0.22 | 28.82 ± 27.44 |
| Pulmonary artery* | 0.88 ± 0.04 | 176.85 ± 110.87 |
| Ribs | 0.75 ± 0.02 | 33.12 ± 12.62 |
| Scapulae | 0.82 ± 0.02 | 14.36 ± 40.87 |
| Skull | 0.89 ± 0.01 | 35.63 ± 83.06 |
| Small bowel | 0.86 ± 0.07 | 32.43 ± 18.87 |
| Spine and Pelvis | 0.90 ± 0.01 | 23.68 ± 32.54 |
| Spleen | 0.94 ± 0.03 | 7.60 ± 7.26 |
| Stomach | 0.91 ± 0.05 | 11.32 ± 9.90 |
| Thyroid | 0.73 ± 0.08 | 7.97 ± 3.13 |
| Trachea | 0.86 ± 0.03 | 10.98 ± 11.17 |
| Ventricle L* | 0.93 ± 0.02 | 5.86 ± 9.34 |
| Ventricle R* | 0.69 ± 0.11 | 8.02 ± 7.41 |

*without fast option (non-available)
