# Supplementary material for: Comparison of organ volumes and standardized uptake values in [18F]FDG‐PET/CT images using MOOSE and TotalSegmentator to segment CT images
Source: Med Phys. 2025 Sep 24;52(10):e70025. doi: 10.1002/mp.70025 (PMC12460933; doi:10.1002/mp.70025)
Supplement: Supplementary file 6 — Supporting information [file MP-52-0-s002.docx]

Table S-6: Evaluation of the impact of the segmentation method (MOOSE and TotalSegmentator) on the SUVmedian of the three anatomical structures with a poor/moderate SUVmean reproducibility.

| **VOI** | ∆_rel_ ≤ 10% | ∆_rel_ ≤ 20% | ∆_rel_ > 20% |
| --- | --- | --- | --- |
| Bladder | 19% | 33% | 67% |
| Lungs | 93% | 98% | 2% |
| Skull | 91% | 100% | 0% |
