# Supplementary figures and images for: Comparison of organ volumes and standardized uptake values in [18F]FDG‐PET/CT images using MOOSE and TotalSegmentator to segment CT images
Source: Med Phys. 2025 Sep 24;52(10):e70025. doi: 10.1002/mp.70025 (PMC12460933; doi:10.1002/mp.70025)

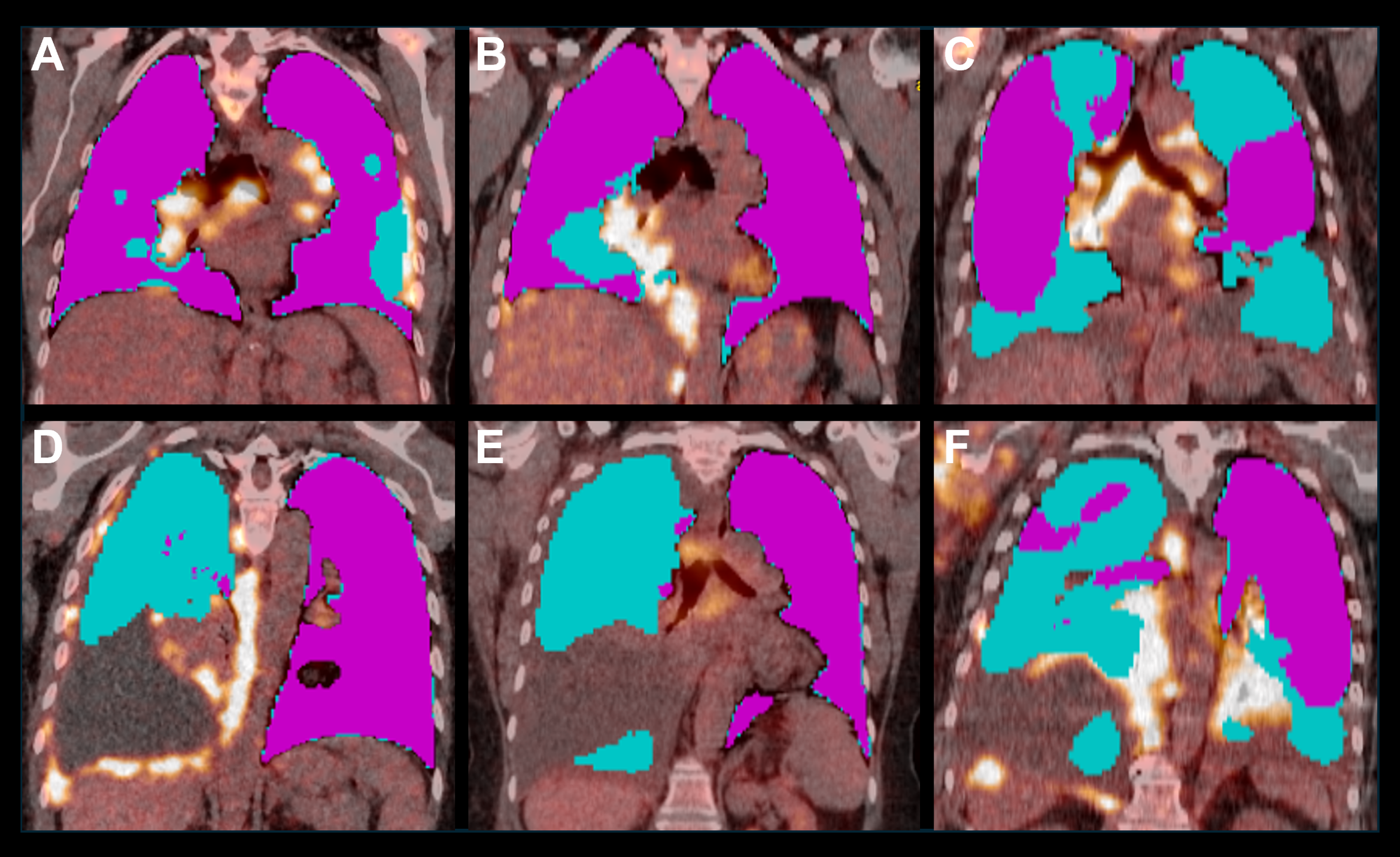

Supplement: Supplementary file 7 — Supporting information [file MP-52-0-s006.tif]
